# Highly Efficient Polydopamine-coated Poly(methyl methacrylate) Nanofiber Supported Platinum–Nickel Bimetallic Catalyst for Formaldehyde Oxidation at Room Temperature

**DOI:** 10.3390/polym11040674

**Published:** 2019-04-12

**Authors:** Fa-Gui He, Bing Du, Gaurav Sharma, Florian J. Stadler

**Affiliations:** 1 College of Materials Science and Engineering, Shenzhen Key Laboratory of Polymer Science and Technology, Guangdong Research Center for Interfacial Engineering of Functional Materials, Nanshan District Key Lab for Biopolymers and Safety Evaluation, Shenzhen University, Shenzhen 518055, China; hepengzhuo@foxmail.com (F.-G.H.); gaurav8777@gmail.com (G.S.); fjstadler@szu.edu.cn (F.J.S.); 2 Department of Optoelectronic Engineering, Shenzhen University, Shenzhen 518060, Guangdong, China

**Keywords:** formaldehyde oxidation, polymeric nanofiber-supported catalyst, platinum–nickel nanoparticles, electrospinning

## Abstract

We fabricated one fibrous-membrane type of flexible and lightweight supported catalyst via loading platinum–nickel nanoparticles (PtNi NPs) directly on the polydopamine-coated polymethylmethacrylate electrospun-fibers (PMMA@PDA). The polymer support with the PDA layer provided numerous active sites, leading to well-monodispersed and sized PtNi NPs on the nanofibers. Through the rational design of PtNi NPs, the resultant catalyst system exhibited 90% conversion for decomposing HCHO (10 ppm) at room temperature with only a low dosage (0.02 g), retaining the high activity for 100 h. This superior catalytic performance stems from the formate oxidation, which was the key intermediate during HCHO decomposition, and was promoted by the existence of a sufficient Pt–OH–Ni interface in the PtNi NPs with an appropriate Pt/Ni ratio of 1:5. Such tailored Pt-based nanoparticles ideally work together with the polymer nanofibers as a support for catalytic reaction. Compared with classical catalysts, our system can handle a comparable efficiency with much lower air resistance and remarkably lower dosage. Furthermore, the membrane-like morphology provides easy handling and minimizes the leaching of catalyst nanoparticles.

## 1. Introduction

As one dominant indoor air pollutant, formaldehyde (HCHO) is quite noxious, even at a very low concentration [1,2,3]. Among the existing approaches, the catalytical oxidation of HCHO into harmless carbon dioxide (CO_2_) and water (H_2_O) at room temperature is widely accepted as the most promising solution due to its environmental friendliness, low energy consumption, and cost saving [4,5,6,7]. In the past decade, a large quantity of work has been carried out to develop the catalyst for promoting the decomposition of HCHO at low temperature. Up to now, the classical catalysts that are applicable to HCHO oxidation under the ambient condition are categorized into two major types: noble metal (e.g., Pt, Pd, Au, and Ag) and metal oxides (e.g., TiO_2_, MnO_2_, and CuO). Generally, noble metal NPs show a higher activity than later oxide-type NPs at room temperature [6,8,9]. Owing to their extremely small size at the nanoscale, a proper support is quite essential and widely utilized to anchor both of these catalysts in order to prevent the loss of the materials. Unfortunately, most of the conventional supports developed for HCHO oxidation are pulverous pellets and beads, which possess high air resistance and are difficult to integrate. To overcome these problems, one kind of structured catalytic support (SCS) strategy has been proposed. Through shaping ceramic or metallic oxide materials into big blocks, e.g., honeycomb-type [10,11], the size of catalysts was efficiently enlarged, leading to much better operability and also improved catalytic properties based on the tailored macrostructure in the supports. Nevertheless, the fabrication of the SCS requires relatively complicated processes. More importantly, the conspicuous frangibility and the relatively large packing density make these supported catalysts difficult to handle in practical applications, especially for indoor air depuration devices. Thus, developing efficient catalysts with superior flexibility, light weight, and easy-going fabrication is still highly desired and attracts lots of attention.

Recently, some researchers attempted to employ the polymeric fibrous membrane or mat as the support for the nanoparticle catalysts and successfully obtained a novel type of flexible catalyst. Besides the remarkable flexibility, three-dimensional (3D) networks with microporous structures possess the additional advantage of low air resistance. Ikegami et al. [12] reported a filter-type catalyst prepared by depositing a ZrO_2_-supported Au catalyst on a commercial poly(ethylene terephthalate) (PET) nonwoven mat, which exhibited good catalytic capability at room temperature with 90% conversion of HCHO to CO_2_ and H_2_O in a batch-type reactor (140 ppm, GHSV 8000 h^−1^) and almost 100% conversion under low concentration (0.5 ppm, GHSV 8000 h^−1^). Similarly, Wang et al. [13] grew MnO_x_ on a polyethylene terephthalate (PET) non-woven mat via in situ synthesis. The HCHO oxidation over the resultant fibrous mat maintained 94% efficiency after 10 h (HCHO concentration of 0.6 mg/m^3^, gas hourly space velocity or GHSV, 17,000 h^−1^, and relative humidity 50%). This improved approach has also been successfully verified and applied to other catalytic reactions [14,15]. 

Nevertheless, to our knowledge, there have been few reports of directly using the polymer as the sole support for a nanocatalyst, although it is supposed to be a very straightforward strategy to fabricate both flexible and lightweight catalysts, which would facilitate easy handling and conventional unitizing in practical indoor air purification. Compared to conventional metal oxide, the major obstacle for polymeric supports is the lack of vacancy sites for oxygen activation at low temperature. Recently, the surface hydroxyl group at the platinum–hydroxyl–M interface (Pt–OH–M, M= Fe or Ni) was verified to have an effective promotion of HCHO oxidation [5,8,16,17,18,19]. In one recent report, Rui et al. [7] fabricated one type of nickel hydroxide-promoted Pt catalysts supported on relatively inert *α*-Al_2_O_3_, over which HCHO conversion reached 99% with a nice stability for more than 100 h at 30 °C. Such superior properties were ascribed to the formation of enormous Pt/Ni(OH)_x_ interfaces promoting HCHO oxidation through oxidizing formate by the abundant associated hydroxyl groups near the Pt active sites. Their work indicated that the tailored Pt-based catalysts containing Pt-hydroxyl (or hydroxide) interfaces have remarkable activity at room temperature, even after being loaded on relative inert supports. 

**Scheme 1:**
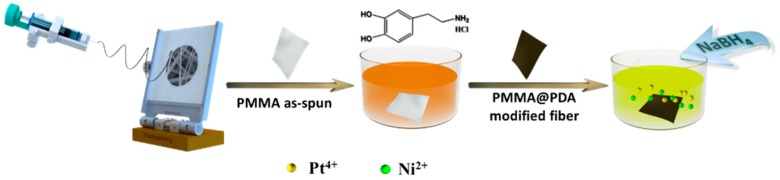
Preparation of platinum–nickel nanoparticles/polydopamine-coated polymethylmethacrylate electrospun-fibers (PtNi/PMMA@PDA) catalytic nanofibrous membrane.

Inspired by this active interface strategy, we herein fabricated a novel supported catalyst, in which flexible and lightweight polymer nanofibers were used as the sole support for the metallic nanocatalyst (Scheme 1). In order to resolve the issue of the polymers’ inert surface property, the platinum–nickel nanoparticles (PtNi NPs) containing active Pt–OH–Ni interfaces were employed to catalytically promote the HCHO oxidation. As the support, the polydopamine-coated poly(methyl methacrylate) nanofibers (PMMA@PDA) were prepared by coating the electrospun PMMA nanofibers via a typical self-polymerization of dopamine. The modified electrospun fibers were found to be favorable for forming monodisperse catalytic particles. With an appropriate composition of PtNi particles, 90% conversion of HCHO with good catalytical stability at room temperature was achieved only using 0.02 g of the manufactured PtNi/PMMA@PDA fibrous membrane. This work developed a promising approach to fabricate a flexible, lightweight, and easy-handling membrane for indoor air purifying.

## 2. Experimental

### 2.1. Materials and Apparatus

Polymethylmethacrylate (PMMA), ammonium hydroxide solution (NH_3_·H_2_O) (A.R., 28 wt % in water), isopropanol (C_3_H_8_O, A.R., 99%), and nitric acid (HNO_3_, A.R., 68%) were obtained from Macklin Reagent Company (Shanghai, China). Chloroplatinic acid hexahydrate (H_2_PtCl_6_·6H_2_O, ACS reagent, ≥37.5% Pt) and dopamine hydrochloride were obtained from Sigma-Aldrich (Hamburg, Germany). *N*,*N*-dimethylformamide (DMF, A.R., 99%), nickel nitrate hexahydrate (Ni(NO_3_)_2_·6H_2_O, A.R., 99%), sodium borohydride (NaBH_4_, A.R., 98%), ethanol, sodium hydroxide (NaOH, ACS reagent, 98%), and hydrochloric acid (HCl, A.R., 36.7%) were purchased from Shanghai Chemical Factory (Shanghai, China). The water used throughout all the experiments was purified with the Millipore system. All the other chemicals were used as received without further purification. 

### 2.2. Preparation of PMMA@PDA Nanofibers

PMMA nanofibers were fabricated by electrospinning 30 wt % PMMA solution in DMF at room temperature with a humidity of ~40%. All the electrospinning processes were carried out on an electrospinning machine from Tong Li Tech Co., Ltd., Shenzhen, China (TL-Pro-BM). The speed of the syringe pump was held constantly at 1.0 mL/h. A voltage of 18 kV was applied, and the distance from the needle tip to the target collector was 15 cm. The received fibers were dried in vacuo at 60 °C for 12 h to remove any residual solvent.

The modification of the PMMA nanofiber surface with polydopamine was performed via a typical self-polymerization of dopamine in water [20,21]. In detail, 0.08 g of a PMMA nanofibrous membrane mat (approximately 10 mm × 10 mm) was immersed into a 35-mL dopamine hydrochloride solution (0.02 M) containing 10 mL of ethanol and 0.5 mL of NH_3_·H_2_O (28 wt % in water). In order to investigate the influence of immersion time on the level of coating, the membranes were kept in the monomer solution for 6 h, 9 h, 12 h, 15 h, and 18 h, respectively. Then, the coated fibers (PMMA@PDA) were flushed with deionized water and 25% aqueous isopropanol solution, followed by freeze drying.

### 2.3. Preparation of PtNi/PMMA@PDA Membrane Catalyst

The deposition of platinum-nickel particles was processed via an immersion method. Approximately 0.08 g of dried PMMA@PDA fibrous membrane (10 cm × 10 cm) was immersed into 50 mL of mixed solution (pH = 6.5, being adjusted by appropriate NaOH) consisting of a certain amounts of H_2_PtCl_6_ and Ni(NO_3_)_2_ with various molar ratios (see in Table 1) for 24 h. Subsequently, metallic particles were formed in aqueous solution with an excessive 100 mL of aqueous NaBH_4_ (0.001 M) solution as the reduction reagent. After being freeze dried, PMMA@PDA fibers loaded with PtNi particles (PtNi/PMMA@PDA) were obtained and stored at room temperature. 

### 2.4. Characterization

The morphologies of the resultant fibrous membrane and the supported nanoparticles were characterized by scanning electron microscope (SEM) equipped with energy-dispersive X-ray spectroscopy (EDS), model SU-70, (Hitachi, Tokyo, Japan) and transmission electron microscopy (TEM, JEM-1230, Nippon Tekno, Japan). The composition of the supported nanoparticles was estimated by testing a solution of the residue of the burned loaded PtNi NPs membrane in nitrohydrochloric acid by inductively coupled plasma optical emission spectrometry (ICP-OES, ICAP-7000, ThermoFisher Scientific, Waltham, MA, USA). Fourier transform infrared spectroscopy (FT-IR, Nicolet 6700, ThermoFisher Scientific, Waltham, MA, USA) and in situ X-ray photoelectron spectroscopy (XPS, PHI 5000 VersaProbe II, ULVAC-PHI, Kanagawa, Japan) were utilized to test the chemical elements on the fibers. In situ diffuse reflectance infrared Fourier transform spectroscopy (DRIFT) was obtained at room temperature using an EQVINOX-55 FFT spectroscope apparatus (Bruker, Karlsruhe, Germany), equipped with a diffuse reflectance accessory and a mercury cadmium telluride (MCT) detector. 

### 2.5. Test of the Catalytic Performance

HCHO oxidation over the catalytic membrane was tested by a homemade fixed-bed reactor (Figure S1). In detail, approximately 0.02 g of the fibrous membrane was compressed and fed into a quartz tubular (diameter 7 mm) under atmospheric pressure at 25 °C. The reactant gas consisted of a simulated air stream (N_2_/O_2_ = 4.80 mL/min) with 10 ppm HCHO and ∼35% relative humidity. Gaseous HCHO was generated by blowing the simulated air through a bubbler containing an HCHO solution (3.5 wt % HCHO). Experiments were performed at a gas hourly space velocity (GHSV) of 24,000 h^−1^. During the reaction, the HCHO concentration in the feed gas ([HCHO]_inlet_) and effluent gas ([HCHO]_outlet_) was determined by the phenol spectrophotometric method. First, 100 mL of HCHO containing a gas stream was passed through a gas washing bottle loaded with a solution of C_6_H_4_SN(CH_3_)C=NNH_2_∙HCl (1 × 10^−4^ wt %, 5 mL). Then, NH_4_Fe(SO_4_)_2_∙12H_2_O (1 wt %, 0.4 mL, dissolved in 0.1 M HCl) was added, and the mixture was left resting in the dark for 15 min. The HCHO concentration was obtained by reading the light absorbance at 630 nm using a spectrophotometer (UV-240, Shimadzu Co., Ltd., Tokyo, Japan) and comparing the data with a standard curve (Figure S2). The standard curve was obtained in a similar manner with the above phenol spectrophotometric method, except for adding a known amount of standard HCHO solution instead of the gas-washing step. The conversion of HCHO was calculated based on the concentration change.

The air resistance of the fibrous membrane and various commercial aluminum oxide pallets (Al_2_O_3_) with different meshes were also tested on the same fixed-bed reactor equipped with a pressure gauge (3051TG, Jiangsu Yiming meter Co., LTD., Yangzhou, China). The feeding gas only consisted of the air steam without any reactive component, i.e., HCHO gas. The pressure drop under different flow rates (20–250 ml min^−1^) was recorded. 

## 3. Result and Discussion

### 3.1. Morphology of PMMA@PDA Support

**Figure 1:**
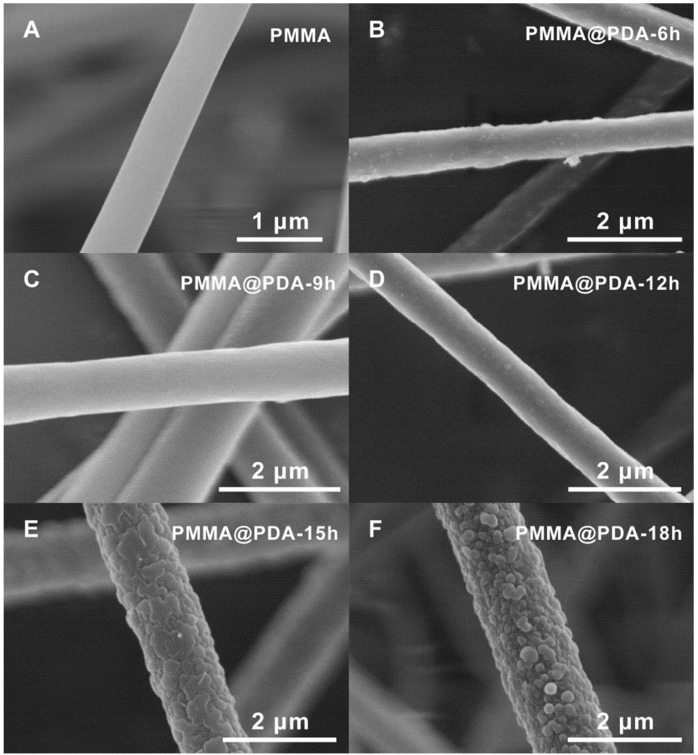
SEM images of: neat polymethylmethacrylate (PMMA) nanofiber (**A**); PMMA nanofiber modified by PDA for 6 h (**B**), 9 h (**C**), 12 h (**D**), 15 h (**E**), and 18 h (**F**).

Figure 1 displays SEM images of neat PMMA nanofibers and the PDA-modified fibers prepared through various coating times. The initial PMMA fibers showed a smooth surface with an average diameter of 600 nm (Figure 1A). In order to introduce the absorbability for metal ions, PMMA nanofibers were further functionalized with PDA (Figure 1B–F). Due to its abundance of amine and hydroxyl groups, PDA was expected to strongly adhere on PMMA surfaces and also to anchor multivalent metal ions [22,23]. As seen in the images, the extent of the modification was found to be closely associated with the immersion time in the dopamine solution, which was the time for dopamine self-polymerization. In view of its impact and smooth PDA layer, PMMA@PDA fibers with an increased diameter of 900 nm prepared by coating for nine hours presented the optimum surface morphology (Figure 1C). Comparatively, when the coating time was less than nine hours, the fibers could not be fully covered by PDA, which is obvious from the conspicuous bare areas and little change of the diameter (Figure 1B). Meanwhile, in the case of the samples with a coating time above nine hours, excessive PDA particles started to appear, forming a rough surface, which might provide the sites leading to the undesired agglomeration of the catalytic particles (Figure 1D–F). Therefore, PMMA@PDA fibers prepared with a coating time of nine hours were selected as support for depositing the catalysts. The successful formation of the PDA layer was also confirmed by FT-IR characterization (Figure S3) with the appearance of a wide band at 3000 to 3500 cm^−1^ corresponding to the phenolic hydroxyl groups and the enhanced band at 1608 cm^−1^ deriving from the aromatic rings [24,25].

### 3.2. Deposition of PtNi NPs on PMMA@PDA and Morphology of PtNi/PMMA@PDA Nanofibrous Membrane

**Figure 2:**
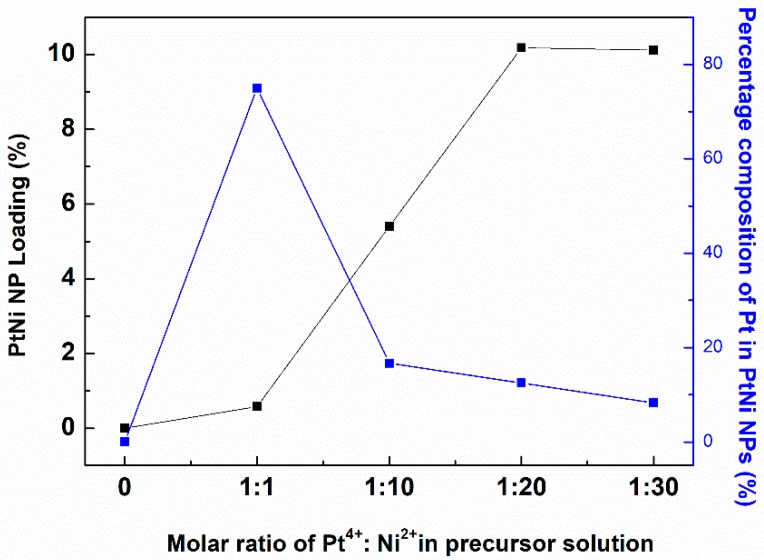
PtNi NPs loading and the percentage composition of Pt in PtNi NPs as a function of the molar ratio of Pt^4+^:Ni^2+^ in the precursor solution.

Form previous reports [19,26], it was demonstrated that the rational composition was one of the crucial factors influencing the formation of the metal–metal oxide interface. Thus, in this work, the PMMA@PDA membrane was immersed in a series of precursor solutions with various molar ratios of Ni^2+^ versus Pt^4+^, respectively, in order to obtain the supported PtNi NPs with different compositions. The compositions of the used precursor solution and the formed PtNi NPs on the fibers were summarized in Table 1. Based on these data, Figure 2 presented the correlation between the molar ratio of Pt^4+^ to Ni^2+^ in the precursor solutions and the one in the resultant PtNi NPs. When Pt^4+^ and Ni^2+^ had the same proportion in the precursor (PtNi/PMMA@PDA-1/1), PMMA@PDA fibers were revealed to be more attractive to Pt^4+^ than Ni^2+^ with 0.53 wt % of Pt, but very few Ni loading. This difference might be explained by the catechol groups on the PDA surface having had a relatively stronger redox interaction with Pt^4+^ than with Ni^2+^, besides having normal complexation, leading to the more stable deposition of Pt sites. After proportionally increasing the concentration of Ni(NO_3_)_2_, the total PtNi loading dramatically ascended. Meanwhile, the Pt composition exhibited a very sharp decrease; in other words, the proportion of Ni was remarkably increased. However, the speedy increase leveled off once the concentration of Ni^2+^ reached to 20 times that of Pt^4+^. 

**Figure 3:**
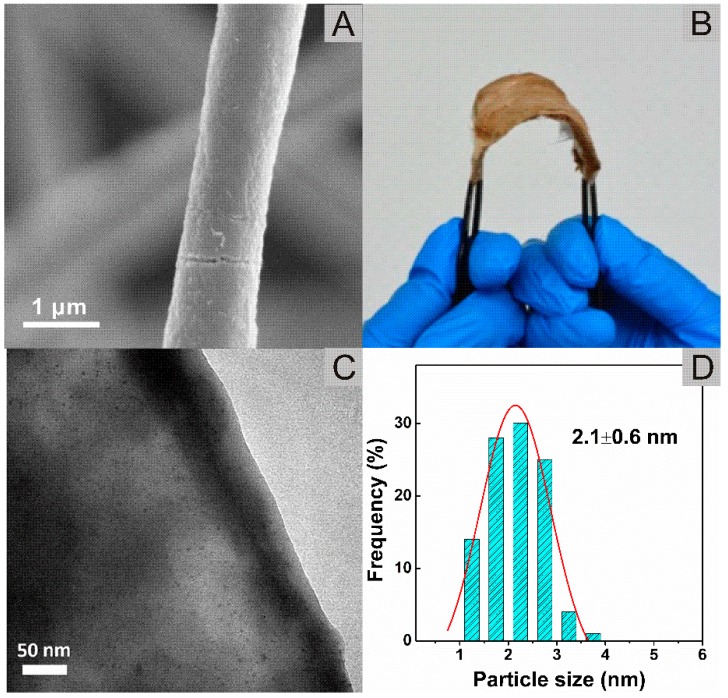
SEM images of PtNi/PMMA@PDA nanofilm (**A**); the digital photo of the PtNi/PMMA@PDA-1/10 nanofibrous membrane (**B**); TEM image of PtNi/PMMA@PDA-1/10 nanofibrous membrane (**C**); Corresponding size distribution of the supported PtNi nanoparticles (**D**).

The morphology of fibrous membrane with the loaded particles were characterized by SEM and TEM as well as by digital photographs of the PtNi/PMMA@PDA-1/10 sample. After loading the catalytic nanoparticles, the PtNi/PMMA@PDA fiber almost kept the same morphology to PMMA@PDA with the same average diameter of 900 nm (Figure 3A). EDS equipped on SEM revealed the homogenous presence of Pt and Ni elements on the fibers (Figure S4). In TEM images (Figure 3B and Figure S5), the monodisperse size distribution of the PtNi NPs was clearly demonstrated, and their average diameter was estimated to be 2.1 ± 0.6 nm (Figure 3C) from 100 particles using ImageJ software. Such uniform size and homogeneous dispersion of PtNi NPs profited from the large number of active sites of electron-rich PDA for depositing ions on the fiber surfaces. Owing to the straightforward deposition strategy without an excessive fragile component (e.g., oxides particles), the composite membrane with supported metal particles presented excellent flexibility, and could be easily bent similar to the conventional polymer electrospun, fibrous membrane (Figure 3D). Such a good flexibility combined with the inherent microporous structure (Figure S6) was expected to improve the chances of the catalyst for the application in the practical air purifying instrument application with low air resistance and easy filter module handling. 

### 3.3. XPS of PtNi/PMMA@PDA Nanofibrous Membrane

**Figure 4:**
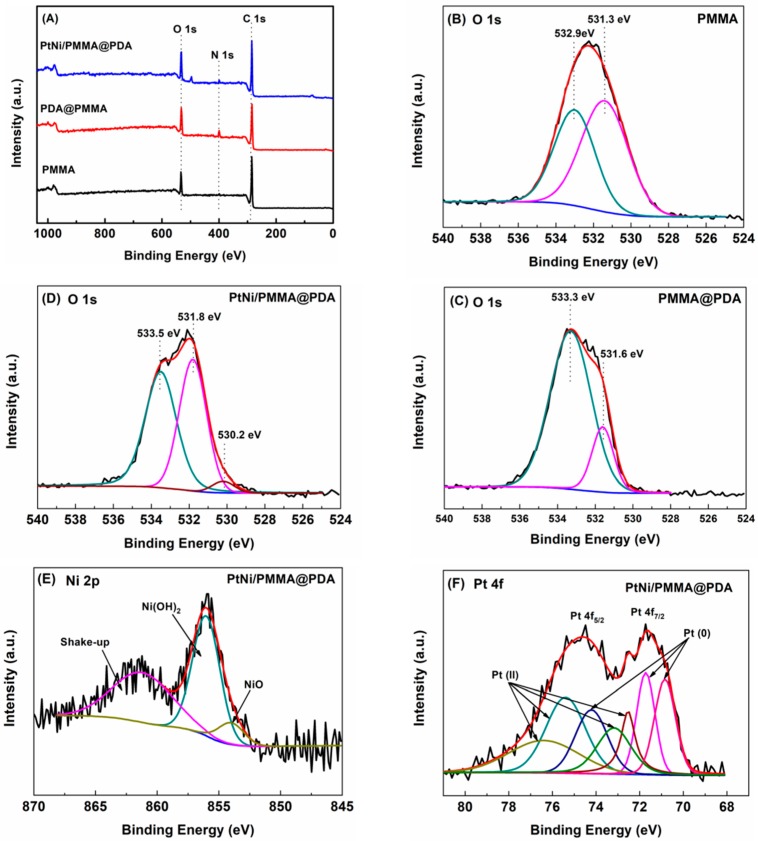
XPS spectrum: Wide survey spectra of the fibrous membranes from each process (**A**); O 1s of PMMA (**B**); PMMA@PDA fiber (**C**) and PtNi/PMMA@PDA (**D**); Ni 2p (**E**) and Pt 4f (**F**) of PtNi/PMMA@PDA fibrous membrane.

The surface elemental composition of the PtNi/PMMA@PDA fibrous membrane was analyzed by XPS, as shown in Figure 4. The typical XPS wide survey spectra of the samples from each process were given in Figure 4A. The appearance of the strong N1s signals in both PMMA@PDA and PtNi/PMMA@PDA samples verified the success of the surface modification with PDA. Figure 4B–D described the high-resolution O1s spectra and their fitted Gaussian peaks of the fibrous mats. Two decomposed peaks locating at 531.3–531.8 eV and 532.9–533.5 eV in the O1s spectra of the polymers (Figure 4B,C) were supposed to be assigned to C=O and C–O groups, respectively [27,28,29,30]. The much higher relative amount of C–O in PMMA@PDA (Figure 4C) than the one in neat PMMA (Figure 4B) should originate from the domination of the hydroxyl in PDA, and further demonstrated the successful coating of PDA outside of PMMA. More interestingly, unlike the spectra of the polymers, three peaks were identified in the case of PtNi/PMMA@PDA (Figure 4D), including a small single peak at 530.2 eV belonging to the bridging oxo groups (–O–) [7]. The appearance of this –O– group was assumed to originate from the existence of NiO or Ni(OH)_2_. Thus, the peaks of C–O in PtNi/PMMA@PDA might not only to be assigned to the hydroxyl from the PDA layer, but also to the Ni(OH)_2_ or NiOOH species. This assumption was further verified by the N2p spectrum of the catalytic fibers. As seen in Figure 4E, the peaks locating at 854.0 eV and 856.2 eV were well-fitted by the signals of NiO and Ni(OH)_2_, respectively [26,31]. In the view of the relative intensities, Ni(OH)_2_ was the predominate component in the Ni sites. Regarding the Pt sites, the binding energies of Pt 4f were 74.78 eV and 71.50 eV because of Pt 4f_5/2_ and Pt 4f_7/2_, respectively [32,33]. After deconvolution, the spectrum of Pt 4f showed the peaks at 74.20 eV, 71.69 eV, and 70.80 eV corresponding to Pt(0), while 76.34 eV, 75.38 eV, 73.12 eV, and 72.50 eV were associated with PtO or Pt(OH)_2_ (Figure 4F) [31,34]. These results revealed the existence of sufficient surface hydroxyl groups from Ni(OH)_2_ neighboring the Pt sites, where the synergy at these formed Pt–OH–Ni interfaces was expected to play a crucial role in facilitating HCHO conversion at room temperature.

### 3.4. Catalytic Properties of PtNi/PMMA@PDA

**Figure 5:**
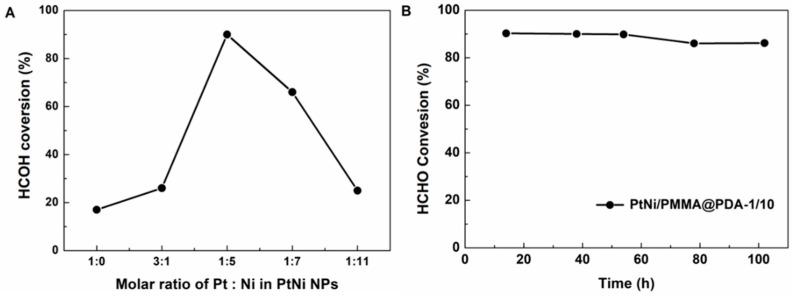
HCHO conversion over various PtNi/PMMA@PDA fibrous membranes with 0.02 dosage at 25 °C as a function of the molar ratio of Pt:Ni in PtNi NPs (**A**); HCHO conversion over PtNi/PMMA@PDA-1/10 as a function of time-on-stream (reactant gas: 10 ppm HCHO, O_2_ 20 vol %, GHSV 24 000 h^−1^) (**B**).

The catalytic property of PtNi/PMMA@PDA was characterized by a homemade fixed-bed reactor at room temperature (25 °C). HCHO conversions over different PtNi/PMMA@PDA samples varying with the Pt:Ni ratio are listed in Table 2. These results suggested a remarkable corresponding effect of the composition of PtNi NPs on its catalytic performance. As shown in Figure 5A, the neat Pt exhibited quite limited activity on the oxidation of HCHO at low temperature. After adding Ni, the supported catalyst displayed a noteworthy enhancement on catalytic efficiency; nevertheless, the activity gradually dropped down, since Ni composition was higher than 84% (i.e., Pt:Ni = 1:5). In the case of PtNi/PMMA@PDA-1/10, the fibrous catalysts performed the optimum catalytic property with an HCHO conversion of 90% and maintained the high efficiency for almost 100 h only with a slight decrease (<5 %). In several reports on Pt-based bimetallic catalysts, the existence of surface hydroxyl groups was accepted to be the key factor to promote HCHO decomposition through formate oxidation at the M–OH–Pt interface (where M is Ni or Fe) [5,7,16,19]. Thus, we assumed that an appropriate composition of PtNi NPs (Ni:Pt = 5:1) provided sufficient surface hydroxyl groups. However, an inferior activity of the catalyst was observed when the Ni percentage composition was relatively high. It might be because the active Pt species were overlaid by the excessive and inert Ni species, leading to a loss of activity. With proper composition, PtNi NPs loaded on fibers also exhibited good stability regarding catalytic performance. As shown in Figure 5B, over the catalytic membrane of PtNi/PMMA@PDA-1/10, HCHO maintained the same conversion efficiency of 90% for more than 50 h, and following that, only a slight decrease to 86% was found after 100 h. On the basis of these results, we might draw a conclusion that with an appropriate composition of metallic components, PtNi NPs on the nanofibers containing a sufficient Pt–OH–Ni interface could possess an efficiently catalytical property of promoting HCHO decomposition at low temperature, which was associated with recent research studies on the Pt-based particles [7,16].

**Figure 6:**
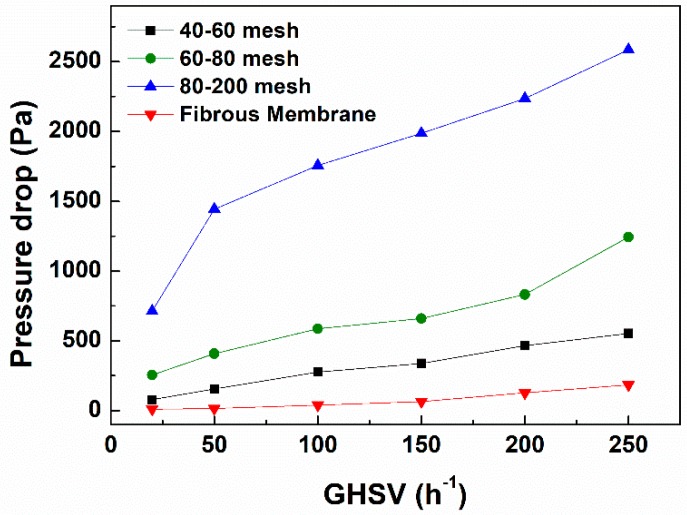
The air resistance of the fibrous membrane and the commercial Al_2_O_3_ with different pellet sizes as a function of the gas flowing speed.

Moreover, using the same instrument but without feeding reactive gas, the air resistance of the composite membrane supporting with PtNi NPs was also investigated in this work and compared with commercial Al_2_O_3_, which is one of the conventional supports for noble metal-based catalysts. As shown in Figure 6, the PtNi/PMMA@PDA membrane had much lower air resistance than the commercial Al_2_O_3_ pellets with various meshes. This result corresponded to and experimentally supported the assertion proposed in the previous literatures that a fibrous-supported catalyst was more effective at reducing the air resistance than the powder-type products [12,35]. Due to this combination of the effective catalytic capability of PtNi NPs and the preferred fibrous morphology with extremely low density, the PMMA@PDA-supported PtNi NPs presented relatively much higher efficiency than the reported catalysts with fibrous supports, especially in view of the weight of dosage of the materials (Table 3), i.e., when normalizing the efficiency on the weight of the catalyst membrane.

### 3.5. In Situ DRIFT

**Figure 7:**
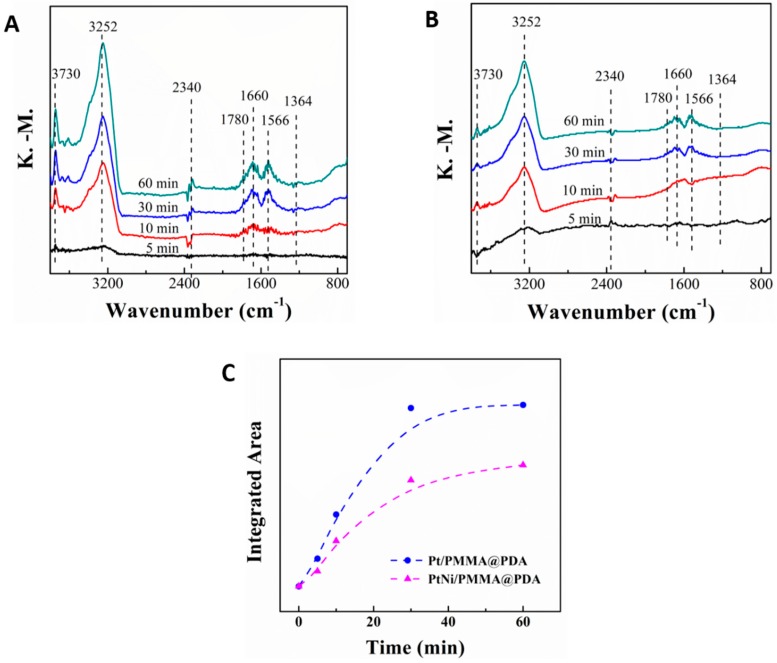
In situ diffuse reflectance infrared Fourier transform spectroscopy (DRIFTS) spectra of gas mixture (O_2_ + He + HCHO) adsorption at 25 ℃ of Pt/PMMA@PDA (**A**) and PtNi/PMMA@PDA-1/10 (**B**); Intensity of 1566 cm^−1^ peak vs. time for Pt/PMMA@PDA and PtNi/PMMA@PDA during gas mixture adsorption for 60 min (**C**).

To investigate the mechanism of the supported catalyst, HCHO conversion over PtNi/PMMA@PDA-1/10, which showed optimal catalytic performance, was characterized by in situ diffuse reflectance infrared Fourier transform (in situ DRIFT) and a Pt/PMMA@PDA without an Ni element was tested too, for comparison. Figure 7 presented the change of the in situ DRITFS spectra of Pt/PMMA@PDA and PtNi/PMMA@PDA as a function of the time in the mixed gas flow of O_2_ + He + HCHO at room temperature. After the catalysts were exposed in the test gases, the bands located at 1364 cm^−1^, 1566 cm^−1^, 1660 cm^−1^, 1780 cm^−1^, 2340 cm^−1^, 3252 cm^−1^, and 3730 cm^−1^ were observed in both of the spectra. According to the previous literature, the bands at 1364 cm^−1^ and 1660 cm^−1^ were ascribed to the asymmetric vibration and symmetric strength of formate, respectively [36,37]. The band at 1566 cm^−1^ was assigned to the surface carbonate from the oxidation of formate over the catalysts [7,37]. Two peaks were at ca. 3734 cm^−1^ and 3252 cm^−1^ corresponding to hydroxyl were attributed to the isolated OH group of formic acid, and the surface OH groups that were bonded to the catalyst overlapped with the groups from the water in air, respectively [30,38]. In addition, the formation of CO_2_ was observed too by the band at 2340 cm^−1^, whereas the signal of CO (the band at 2042 cm^−1^) was not observed [7,37]. Hence, it was suggested that HCHO conversion over these two catalysts both followed a pathway of the direct formate oxidation. This result corresponded with the mechanisms reported in several literatures about the NaBH_4_-reduced noble-metallic catalyst [39] or the alkali metal-promoted catalyst for HCHO deposition [5]. It was worth noting that the absorption of formate over PtNi/PMMA@PDA was found to be noticeably lower than the one of the Pt/PMMA@PDA sample. Since the destruction or oxidation of formate has been widely accepted as the critical section in the oxidation of HCHO, the calculation of the integrated formate species band at 1566 cm^−1^ was analyzed to investigate the different activity between the neat Pt sample and the PtNi sample [5,7]. Figure 7C shows the curves of the intensities of formate vibration bands at 1566 cm^−1^ as a function of the reaction time. The intensities of the bands of both catalysts ascended and gradually stopped rising. Importantly, the relative amount of accumulated formate species over PtNi/PMMA@PDA was always lower than the one over the supported neat Pt, and the difference increased over the time, demonstrating the higher activity of PtNi/PMMA@PDA. Moreover, the band corresponding to the isolated OH groups from the formic acid in the spectra of PtNi/PMMA@PDA was also considerably smaller than the one in the case of Ni/PMMA@PDA, which further identified the faster conversion of formate over the PtNi/PMMA@PDA catalyst. Hence, it’s reasonable to deduce that the deposition of HCHO over PtNi/PMMA@PDA followed the pathway of direct oxidation of formate and the existence of an Ni species, which provided the surface hydroxyl groups, and an active interface of Pt–OH–Ni played a key role in removing HCHO through its promotion of the oxidation of formate into CO_2_ and H_2_O.

## 4. Conclusions

Through introducing PtNi NPs containing sufficient surface hydroxyl groups, we fabricated a novel PtNi-supported catalyst directly on PMMA@PDA nanofibers. Thanks to the strong effect of the Pt–OH–Ni interface on promoting formate oxidation, the obtained PtNi/PMMA@PDA nanofibrous membrane achieved a high efficiency (~90%) with good flexibility and longtime stability as well as low air resistance for HCHO decomposition at room temperature only using a dosage of 0.02 g. This pioneering work further demonstrates the prominent effect of Pt–metal hydroxides interface on the catalytic oxidation of HCHO at low temperature, and opens a new window to design a flexible, lightweight and easy-handling catalyst for practical indoor air purification.

## 5. Patent

A patent application for the fabrication of PtNi/PMMA@PDA nano-fibrous membrane was submitted as International Application numbers PCT/CN2018/116866.

## Figures and Tables

**Table 1:** Composition of the precursor solution and the deposited PtNi NPs on PMMA@PDA fibers.

Sample ID	Precursor Solution ^a^			Deposition of PtNi NPs ^b^			
	Content (mM)		Pt^4+^:Ni^2+^	Loading Capacity (wt %)^-^			Pt:Ni
	Pt^4+^	Ni^2+^		Pt	Ni	PtNi NPs	
PtNi/PMMA@PDA-1/1	0.494	0.494	1:1	0.53	0.05	0.58	3:1
PtNi/PMMA@PDA-1/10	0.494	4.940	1:10	2.16	3.24	5.40	1:5
PtNi/PMMA@PDA-1/20	0.494	9.880	1:20	3.19	6.99	10.18	1:7
PtNi/PMMA@PDA-1/30	0.494	14.82	1:30	2.4	7.72	10.12	1:11
Pt/PMMA@PDA-1/0	0.494	0.000	1:0	0.23	---	0.23	---
Ni/PMMA@PDA-0/10	0.000	4.940	0:10	---	5.95	5.95	---

^a^ The content and molar ratio of Pt^4+^ and Ni^2+^ in aqueous solution; ^b^ Weight percentage content and molar ratio of Pt and Ni in PtNi NPs were estimated by testing concentrations of Pt^4+^ and Ni^2+^ in the residue solutions of the burned membrane dissolving in nitrohydrochloric acid via inductively coupled plasma optical emission spectrometry (ICP-OES).

**Table 2:** HCHO conversion over the catalytic membrane with different PtNi NP compositions.

Sample ID	Molar Ratio of Pt:Ni in PtNi NPs	HCHO Conversion(%)
PtNi/PMMA@PDA-1/1	3:1	26%
PtNi/PMMA@PDA-1/10	1:5	90%
PtNi/PMMA@PDA-1/20	1:7	66%
PtNi/PMMA@PDA-1/30	1:11	25%
Pt/PMMA@PDA-1/0	1:0	17%
Ni/PMMA@PDA-0/10	0:10	<5%

**Table 3:** Comparison of the HCHO oxidation efficiency over the catalyst per gram per hour.

Sample ID	Type of Reactor	HCHO Conc. (ppm)	Flow Rate (mL/min)	Volume of Catalyst (mL)	Weight of Catalyst (g)	GHSV (h^−1^)	HCHO Oxidation Efficiency (mg_HCHO_ g^−1^ h^−1^)	Ref.
PtNi/PMMA@PDA	Fixed bed reactor	10	100	0.25	0.02	24 000	3.75	This work
Au/ZrO_2_/PET	Fixed bed reactor	0.5	200	1.5	0.3	8 000	0.025	Ref. [12]
MnOx/PET	Fixed bed reactor	0.48	1000	3.5	0.5	17 000	0.072	Ref. [13]

## Supplementary Materials

The following are available online at https://www.mdpi.com/2073-4360/11/4/674/s1.
