# Supplementary material for: Highly Efficient Polydopamine-coated Poly(methyl methacrylate) Nanofiber Supported Platinum–Nickel Bimetallic Catalyst for Formaldehyde Oxidation at Room Temperature
Source: Polymers (Basel). 2019 Apr 12;11(4):674. doi: 10.3390/polym11040674 (PMC6523936; doi:10.3390/polym11040674)
Supplement: Supplementary file 1 [file polymers-11-00674-s001.pdf]

Electronic Supporting Information (ESI)

Highly efficient polydopamine coated polymethylmethacrylate nanofiber supported platinum-nickel bimetallic catalyst for formaldehyde oxidation at room temperature

Fa-Gui Hea, b, Bing Dua,, Gaurav Sharmaa, b, Florian J. Stadlera*

a College of Materials Science and Engineering, Shenzhen Key Laboratory of Polymer Science and Technology, Guangdong Research Center for Interfacial Engineering of Functional Materials, Nanshan District Key Lab for Biopolymers and Safety Evaluation, Shenzhen University, Shenzhen 518055, PR China

b Department of Optoelectronic Engineering, Shenzhen University, Shenzhen 518060, Guangdong, China.

* E-mail: dubing@szu.edu.cn

Table of Contents

Figure S1. Scheme of the home-made instrument for the test of HCHO conversion and the air resistance over the catalyst	S2
Figure S2. Standard curve of the HCHO conversion detected by UV accessory	S2
Figure S3. FT-IR spectrum of PMMA, PMMA@PDA and PtNi/PMMA@PDA	S3
Figure S4. EDS elemental mapping and spectrum of PtNi/PMMA@PDA-1/10	S4
Figure S5. TEM image of PtNi/PMMA@PDA nano-fibrous membrane with relative low magnification	S5

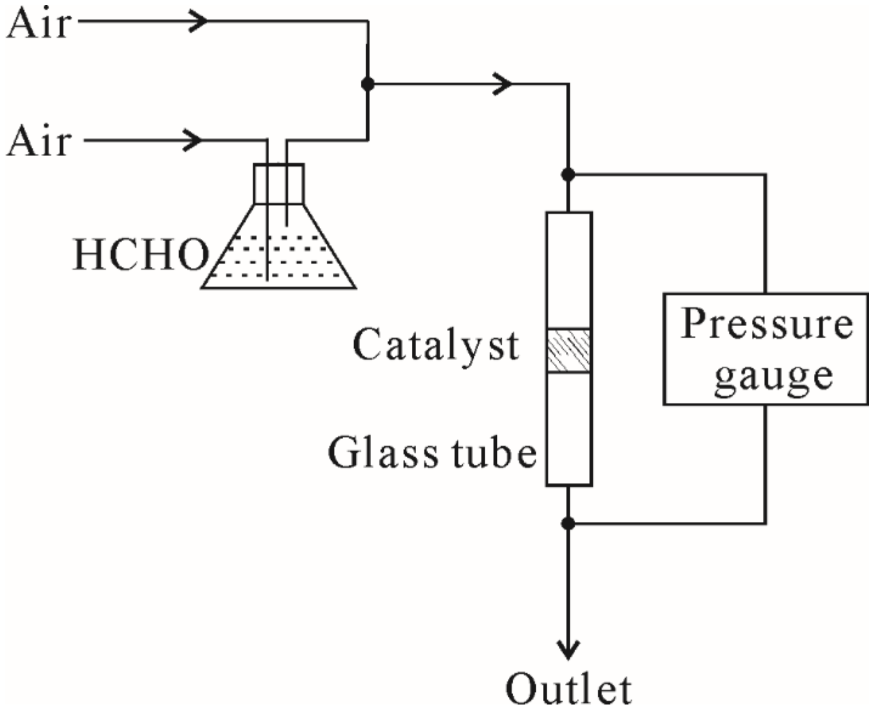

Figure S1. Scheme of the home-made instrument for the test of HCHO conversion and the air resistance over the catalyst

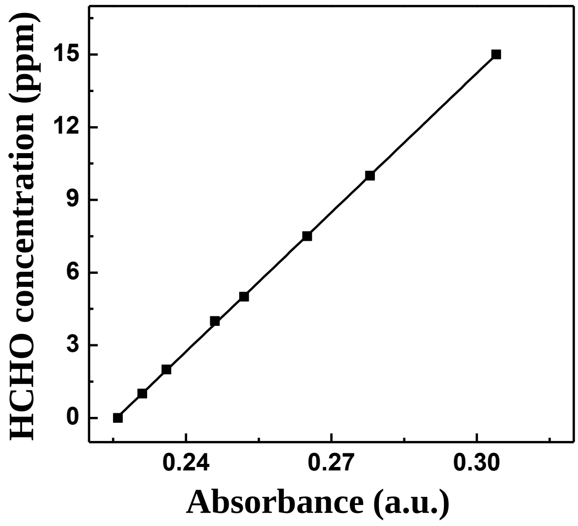

Figure S2. Standard curve of the HCHO conversion detected by UV accessory

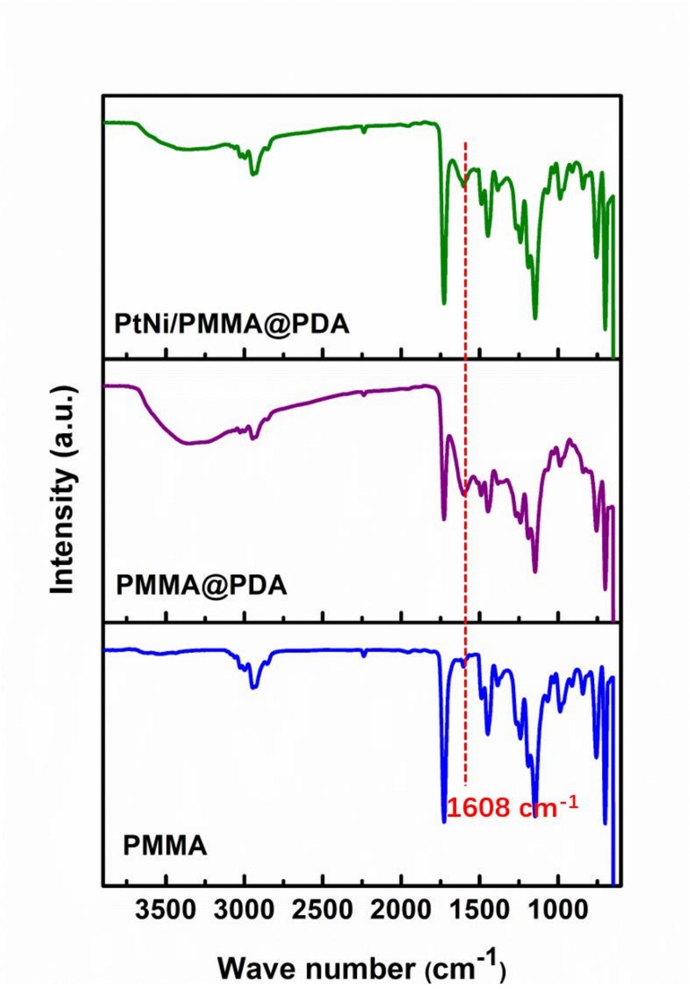

Figure S3. FT-IR spectrum of PMMA, PMMA@PDA and PtNi/PMMA@PDA

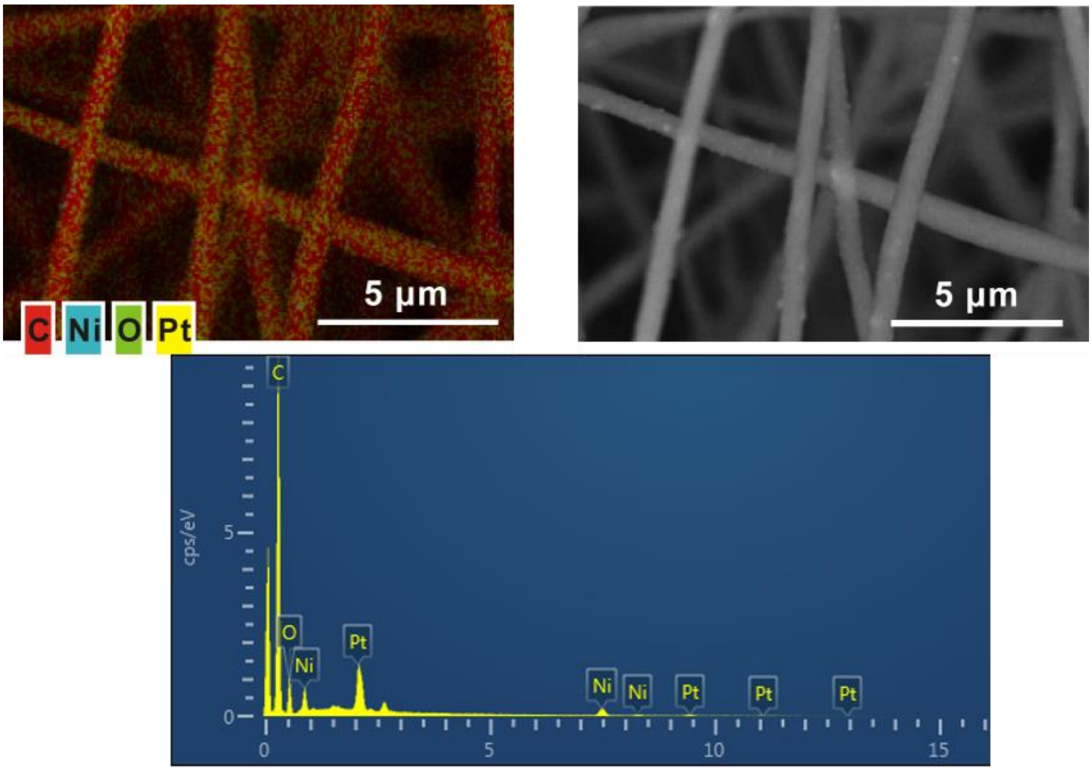

Figure S4. EDS elemental mapping and spectrum of PtNi/PMMA@PDA-1/10

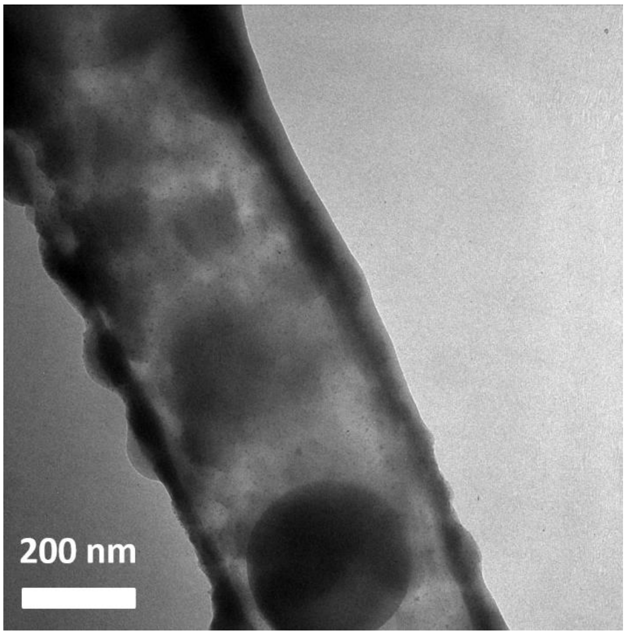

Figure S5. TEM image of PtNi/PMMA@PDA nano-fibrous membrane with relative low magnification
